# Congruence in European and Asian perception of Vietnamese facial attractiveness, averageness, symmetry and sexual dimorphism

**DOI:** 10.1038/s41598-023-40458-1

**Published:** 2023-08-16

**Authors:** Ondřej Pavlovič, Vojtěch Fiala, Karel Kleisner

**Affiliations:** Department of Philosophy and History of Science, Faculty of Science, Charles University, Vinicna 7, 128 44 Prague, Czech Republic

**Keywords:** Psychology, Evolution, Psychology and behaviour

## Abstract

Attractiveness is a proposed universal cue to overall biological quality. Nonetheless, local raters and raters of the same ethnicity may be more accurate in assessing the cues for attractiveness than distant and unfamiliar raters. Shared ethnicity and shared environment may both affect rating accuracy: our aim was to compare their relative influence. Therefore, we photographed young Vietnamese participants (N = 93, 33 women) from Hanoi, Vietnam. The photographs were rated by Czechs, Asian Vietnamese, and Czech Vietnamese (raters of Vietnamese origin who lived in Czechia for all or most of their life). Using geometric morphometrics, we measured facial shape cues to biological quality: averageness, asymmetry, and sexual dimorphism. We expected that Vietnamese raters residing in Czechia and Vietnam would agree on perceived attractiveness and use shape-related facial cues to biological quality better than Czech European raters, who are less familiar with East Asians. Surprisingly, mixed-effect models and post hoc comparisons identified no major cross-group differences in attributed attractiveness and path analyses revealed that the three groups based their rating on shape-related characteristics in a similar way. However, despite the considerable cross-cultural agreement regarding perceived attractiveness, Czech European raters associated attractiveness with facial shape averageness significantly more than Vietnamese raters.

## Introduction

During the Communist era, people from various socialist countries moved to the Central European socialist states as a result of state-regulated migration. For example, thousands of Vietnamese citizens took part in a state-funded labor training and exchange program in Central Europe^1^. After the collapse of the Communist regimes in the early 1990’s, some Vietnamese immigrants returned to Vietnam, while many others remained in Central European countries and established themselves as small traders^2,3^. In subsequent years, more Vietnamese individuals came to the Czech Republic seeking better economic prospects, and their children, who grew up in the Czech society, often have an ambivalent connection to their Vietnamese roots and culture. They grew up among Czech children, often had Czech caregivers^4^, their Czech is usually at a native speaker level, they study at Czech universities and are surrounded by Czech friends and sometimes Czech partners^5^. They identify themselves as the "banana generation" (as they themselves say: “yellow on the outside, white on the inside”), feeling more Czech than Vietnamese. In that respect, they often radically differ from their parents’ generation^6,7^.

Nowadays, there are over 63,000 registered Vietnamese citizens who live in Czechia. They form the third largest minority after Slovaks and Ukrainians^8^. Together with the Vietnamese who have a Czech citizenship, they are by far the most common visually distinct (i.e., non-European looking) group in the otherwise relatively ethnically homogenous Czech society. As such, they may present an ethnic group that is despite the visually distinct appearance quite familiar to Czechs of European origin. It is thus possible that Czechs of European origin are, in terms of social perception, capable of processing Vietnamese faces almost as well as (European) Czech faces. On the other hand, while the Czech majority is aware of people of Vietnamese origin living among them, they still constitute a relatively small fraction of the Czech society. European Czechs may only know a few persons of Vietnamese origin, which could reduce their ability to process Vietnamese faces.

### Facial perception

The human face contains a lot of information regarding basic personal characteristics, such as gender, age, and ethnic background. It also provides cues to the mood, attention, health, social status, and various personality traits of the bearer^9–14^. After seeing a face for even a fraction of a second, a perceiver can assess traits such as attractiveness^15^, trustworthiness^16^, or aggressiveness^17^. Moreover, these assessments do not substantively change with a longer exposure to that face^15^. There is thus convincing evidence that the human face plays a pivotal role in social interactions and in the formation of first impressions.

Although humans can recognise thousands of individual faces and assess their attributes, there is good evidence to the effect that people are better at processing faces of their own race than the faces of other, visually distinctive, races^18–20^. Raters thus have a higher processing ability in their own population; we refer to this ability using the term ‘perceptual expertise’ and shall therefore speak about a ‘perceptual expertise hypothesis’. If it is correct, reported cross-cultural disagreement on various perceived facial characteristics, such as attractiveness or trustworthiness^21–23^, is the result of a relatively lower perceptual expertise, primarily due to lack of exposure to and experience with processing of faces of other ethnicities^24^. Basic facial encoding schemes develop during the first years of life—mostly through exposure and visual experience^25–28^. Frequent early exposure to faces of visually distinctive populations can allow children to acquire a similar level of perceptual expertise regarding faces from all these distinctive populations^29–31^. Evidence suggests that learning and visual experience can have this effect also in adults^24^. Of equal importance is the fact that differentiation among individual faces^32,33^, trait attribution^34^, and behaviour towards group members^35^ are strongly influenced by individual motivation^36^ as well as ingroup/outgroup classification, possibly based on phenotypical differences and ethnicity^37^. As a result, raters from the majority population lacking such motivation may perceive ethnic minorities in a stereotypical way and feel that ‘they all look alike’^38^.

What remains unclear is the relative contribution of various factors and processes, such as one’s own environmental influence or previous exposure and experience, to the formation of judgements and preferences based on facial perception.

### Attractiveness

One of the perceived facial characteristics affected by perceptual expertise is attractiveness. Evidence suggests that members of groups that are isolated from each other do not agree on facial attractiveness ratings^21,22,39^. This is significant because attractiveness plays an essential role in mate choice and other social interactions: attractive people tend to be attributed more positive personality traits^40–42^, they are seen as healthier^43^, more intelligent^44^, and they are more likely to be hired for a job^45^. In the context of mate choice, attractive people have more dates, are seen as more competent in dating, and their dating partners report higher satisfaction with their dates^46–48^. While this might be due to the attractiveness halo effect^49^ and lack any other function, it may also stem from attractiveness perception being an adaptation. Evolutionary psychology proposes that attractive traits function as cues to various aspects of underlying biological quality^43^, and that is why they are preferred.

In the East Asia, ideal woman is youthful, cute and innocent. The most prominent features associated with beauty are the size of an individual’s eyes and pale white skin without irregularities or visible defects; preferences for less prominent chin and cheekbones are also reported^11,50–52^. Large and pale eyes are believed to resemble features of all baby mammals. Such configuration, also known as baby schema, is generally perceived as cute^53–55^, and makes an individual look younger. This may explain why one of the most common aesthetic procedures in Asia is blepharoplasty, correcting eyelid defects^56^.

In most human populations across the globe, including Asian societies, pale white skin is historically associated with high social status, as lower class often had tanner skin from working in the fields^57^. Due to their desire for spotless, clean, fair, white skin, Asian women often tend to limit exposure to sunlight by using long sleeves, protective sunscreen, and by staying in the shade. However, this leads to higher incidence of Vitamin D deficiency in East Asian women^58^.

Although these beauty ideals are likely deeply rooted in Asian history and culture, the Eurocentric influence is also a crucial factor^52^. Both in Western and Asian media, a lot of attention is given to people of mixed origins who exhibit European features with Asian appearance, also known as “pan-Asians” due to cosmopolitanism and homogenization of various Asian ethnicities^59^. Moreover, Asian women are more likely to endorse mainstream beauty standards similar to white women, leading to higher rate of body dissatisfaction among East Asian women^60,61^. This tendency for self-comparison to the Western standarts speaks in favor to the idea that East Asians are generally more susceptible to universal sociocultural norms, independent of kinship^62^ Averageness (proximity to the population norm), facial symmetry, sexual dimorphism, age, and cues to body mass were described as the most important shape-derived traits that affect facial attractiveness^46^. In the following, we review their contribution to overall attractiveness and their proposed evolutionary significance.

Averageness of face shape (in the sense of low deviation from the population mean) is perceived as attractive and healthy-looking^63–66^. Some studies suggests that averageness of the face shape indicates higher heterozygosity, genetic diversity and immunocompetence, and therefore also a higher biological quality^43,64,67,68^. Nonetheless, it is crucial to refrain from assuming direct and oversimplified linear relationships between genetic diversity and the benefits of genetic heterozygosity, as well as between averageness of face shape, heterozygosity, or facial attractiveness. For instance, research based on populations in Iceland and Denmark reveals an n-shaped curve in the bivariate association between fertility and the degree of kinship within couples, suggesting that genetic diversity is beneficial only to a certain degree. This implies that at least in some populations the reproductive success and fertility rates may rise as the level of kinship increases, with a steep decline among couples who are second cousins or have even closer kinship^69,70^. The association between higher face shape averageness and attractiveness is also not linear: Evidence indicates that while averageness is attractive, the most attractive faces are not the most average ones^63,65,71^, which points to the multifaceted nature of human facial attractiveness. Altogether, while face shape averageness exhibits a certain degree of preference across various human populations, some uncertainty still surrounds this phenomenon. Studies based on cross-cultural ratings of mutually isolated groups point to the importance of visual experience with and/or spatial proximity between individuals of the rating and rated population^72,73^. Other studies stress the importance of familiarity with the given stimuli^74,75^, providing an explanation that is relatively independent of facial cues regarding biological quality.

It has been suggested that facial symmetry is an indicator of biological quality^76–78^. Fluctuating asymmetry (random deviation from bilateral symmetry) is considered to be a measure of an organism’s relative developmental instability and is known to increase under environmental and genetic stress^77,79–81^. Moreover, lower fluctuating asymmetry is linked with higher intelligence^82^, higher assessment of perceived health^83^, and higher facial masculinity in men^84^. More symmetrical faces are also attributed more positive personality attributes^85,86^. It has been shown that in men, but not in women, facial symmetry is weakly but credibly associated with facial attractiveness^66^. All in all, reports on the relationship between facial symmetry and attractiveness are rather mixed: according to some studies, lower fluctuating asymmetry is linked with higher attractiveness^66,87,88^, while other studies found no such effect^72,89,90^. This inconsistency of findings could be due to differences in methodology or perhaps overestimation of effect sizes due to publication bias^91^. Some studies worked with unmanipulated facial photographs, while other studies used images that were artificially manipulated using various manipulation techniques^41^.

Sexual dimorphism in human faces emerges around puberty and is the consequence of the increasing effect of sex steroids^92,93^. In the faces of women, feminine facial traits are associated with higher perceived attractiveness within and across human populations^43,94^ and often interpreted as reliable cues to fertility and reproductive capacity^95–97^.

In the case of male faces, the situation is less clear. Some studies report a preference for more masculine facial configurations^98–100^, others found preference for less masculine, i.e., more feminine faces^101–103^, and yet other researchers found no preference for either masculine or feminine facial traits^104,105^. Such highly mixed results could be partly due to methodological differences in stimuli preparation^43^, but different environmental and socioeconomic conditions^106,107^ might also drive some systematic shifts in preferences for facial masculinity across populations^72,108,109^. Moreover, interpersonal differences between female raters related to hormone levels, phase of the menstrual cycle, and relationship status might also affect their masculinity preferences^110,111^.

Masculine traits are interpreted as a signal of good health^67^. They are associated with higher perceived dominance^112^, higher social status^113^, and good fighting ability^114^. While these characteristics are preferable, masculinity is also a cue to aggressiveness^114,115^ and low partner fidelity^116^, that is, characteristics potentially detrimental to the success of a partnership. Women thus potentially make a trade-off between desirable and detrimental characteristics of masculine males^117^, which is why the results of studies on this subject differ: the optimal balance varies depending on both environmental conditions and women’s individual characteristics.

Age negatively affects perceived attractiveness, especially in women. Age-related attractiveness decline seems stable across various human populations^118,119^. Although age serves as a potential cue to residual fertility in women^120^, the use of hormonal contraception largely nullifies this effect^97^. In men, the age-related decline of attractiveness is much slower and can be partly compensated by simultaneous raise in perceived power and dominance^118,121^. Despite the age-related attractiveness decline, older individuals, in particular women, report on average higher self-perceived attractiveness^122^ and individuals of both sexes rate faces of all ages in more balanced manner that younger participants, who tend to rate younger faces as more attractive than old faces^123^.

Relative body weight also affects facial features and, in turn, the ascribed characteristics. It has been shown that people can estimate BMI from facial cues alone^124^. Facial cues to relative body mass, mainly facial adiposity, influence perceived attractiveness and health, although the level of facial adiposity that is considered most attractive and most healthy can slightly vary between populations^125–127^.

In our previous study on 'Central European facial attractiveness,' we made a noteworthy discovery indicating that a shared environment can generate a consensus on perceived facial characteristics, thereby diminishing the influence of one's ethnicity^128^.

### The current study

Our present study aims to further examine the role of shared environment and ethnical background on the same populations, namely Czechs, Czech Vietnamese (members of the Czech population of Vietnamese origin) and Asian Vietnamese, with Vietnamese faces as stimuli. This dual perspective approach may offer new insights into facial perception among immigrants or other bicultural individuals.

We collected photographic facial stimuli of ethnically Vietnamese persons and had them rated by Asian Vietnamese (AVN), Czech Vietnamese (CZVN), and Czech European raters (CZE). Our aim was to see whether the ratings of these three groups converge. The Czech Vietnamese represent a minority that is both encultured into and phenotypically distinct from the local population of European origin. This makes the Czech Vietnamese a suitable group for investigating the influence of varying level of inaccuracy in facial attractiveness attribution, which may be due to different levels of familiarity with given facial stimuli.

We suggest that Czech raters of European origin are the least familiar with Vietnamese faces, Asian Vietnamese are most familiar with them, while Czech Vietnamese are potentially somewhere in-between the two groups. Based on this, we propose the following hypotheses:

In assessing Vietnamese faces, Vietnamese raters might acknowledge the characteristics which do serve as cues to biological quality but are not noticed by ethnically European perceivers. On the other hand, people of Vietnamese origin who grew up in Czechia surrounded by few Asian and many European faces may judge the target faces in a way that is more similar to the Czech European than the Asian Vietnamese perspective. That would imply that perceptual schemes are based on prevailing visual diet even when the perceived faces are phenotypically different^29^ and may be locally adaptive. Under this assumption, raters residing in Europe (Czech Republic) should, regardless of their ethnic origin, assess the attractiveness of Vietnamese faces by ‘European optics’, which differs from ‘East Asian optics’ and reflects the demands of local social and environmental factors (hence a hypothesis regarding ‘socio-environmental factors’).

Alternatively, it is possible that the Czech Vietnamese maintain largely the same preferences as Asian Vietnamese raters, following patterns and adaptations established in East Asian Vietnamese population. These preferences, typical for Asian (Vietnamese) raters, are thus not affected by European socio-environmental conditions. In that case, Czech European raters might judge attractiveness differently. Insufficient exposure to Vietnamese faces may lead Czech European raters to application of the same perceptual schemes as they use for European faces, which could result in overlooking adaptive cues to biological attractivity. If the above is the case, ratings of all Vietnamese raters (from both the Czech Vietnamese and Asian Vietnamese sample) should converge and both should differ from the ratings made by European Czechs. This would amount to support of the hypothesis of ‘parental impact effect’ in favour of own population.

The Asian Vietnamese are not affected by Czech culture and environment, nor are their preferences for Vietnamese faces shaped by it. On the other hand, it is possible that Czech European raters may trace attractiveness cues equally well as the two Vietnamese groups. In this case, a comparison of ratings by the three groups would reveal no effect of rater population on the attribution of a facial characteristic (thus supporting the hypothesis of a ‘cross-population agreement’).

Finally, it is possible that differences in the visual diet and other socioenvironmental factors between the three studied populations could be of such magnitude that their preferences could completely diverge, resulting in disagreement in their ratings (‘disagreement hypothesis’).

Furthermore, we investigated the relative contribution of three objectively measured facial traits—face shape averageness, facial asymmetry, and sexual dimorphism of facial shape—to perceived facial attractiveness across the three groups. While more symmetrical, more average, and more sex-typical configurations (male-like in men, female-like in women) should be generally preferred (see above), the three rating groups may differ in their cue utilisation. There are substantial differences in the magnitude of face shape sexual dimorphism across various populations^129^. In particular, Asian faces are characterised by a lower level of face shape sexual dimorphism than European faces^130^ (see also Fig. 2). Perceivers of Vietnamese origin may thus rely more on other shape-related facial cues, for example facial averageness and symmetry. This may render, at least for the Asian and Czech Vietnamese groups, face shape sexual dimorphism effectively irrelevant with respect to perceived attractiveness.

## Materials and methods

### Data acquisition

We took facial portraits of 93 Vietnamese participants (60 men, average age 21.1 years; SD = 1.85, range 18–33 years; and 33 women, average age 21.8 years, SD = 4.21, range 18–40 years). The data were collected in Hanoi, Vietnam, during several sessions between 25 January 2018 and 7 February 2018, always in the same room and under the same standardised conditions. Informed consent was obtained from all participants via computer screen prior to participation in the data collection.

Facial portraits were taken using a standardised procedure^131^ used in a previous study^128^. Participants were instructed to avoid any facial makeup or jewellery and were given a black t-shirt to exclude any influence of their clothing. They were seated on a chair without a back rest, in front of a white background, and were instructed to sit straight, look directly at the camera, and adopt a neutral facial expression. Photographs were taken from a tripod set to match the sitting height so as to keep the target’s face in the middle of the frame. To preserve natural variability in facial size, the distance between the lens and the tip of target’s nose was always set to 125cm. This distance allowed us to obtain the sharpest possible picture with the 50mm lens used.

We used a colour camera Canon 60D connected to a studio flash and equipped with a Canon RF 50mm STM lens. The focus point was set to the left eye. Exposure was set to ISO 100, shutter speed 1/100, aperture f/8, and 2/3 of strobe power. Portraits were shot into uncompressed raw files (*.CR2 format) and later processed to JPEG files in sRGB colour space. White balance was corrected and colour correction patch (X-Rite Color Checker) was photographed at the beginning of each session to enable subsequent correction and processing of photographs.

All sampling and experimental procedures conformed to current institutional, national, and international guidelines as well as the Helsinki Declaration. This study does not include information that could lead to the identification of any particular participant. All procedures were approved by the Institutional Review Board of the Faculty of Science of the Charles University (protocol ref. number 04/2020).

### Rating of facial images

The stimuli were assessed for attractiveness by an unrelated sample of Asian Vietnamese, Czech Vietnamese, and Czech Europeans. Raters of each sex rated only portraits of the opposite sex. Some raters did not finish the whole rating session—thus the range in the number of raters. Facial photographs of Asian Vietnamese women were rated by 81–86 Asian Vietnamese men (mean age = 22.2; SD = 3.76; range = 18–47), 46–47 Czech Vietnamese men (mean age 24.2; SD = 2.98; range = 18–33), and 64 Czech European men (mean age = 24.0, SD = 5.13, range =19–43). Facial photographs of Asian Vietnamese men were rated by 116–124 Asian Vietnamese women (mean age = 22.96; SD = 4.26; range = 18–48), 63–67 Czech Vietnamese women (mean age = 23.75; SD = 5.25; range = 18–55) and 97–104 Czech European women (mean age = 25.22; SD = 5.31; range = 18–47).

Raters viewed each portrait on a computer screen in a browser with survey session set to full screen by default. It displayed one portrait at a time, centred to the middle of the screen, in a randomised order for each session, and no time limit for the exposure of each image. Raters assessed facial attractiveness on a 7-point Likert scale (1 being ’very unattractive’ and 7 ‘very attractive’). If raters recognised the stimulus person, they were also instructed to click on ‘I know this person’ button to skip rating the current image.

Raters were recruited via internet (social media platforms) or asked personally and redirected to an online survey platform (Qualtrics.com). Participants younger than 18 and older than 50, those of non-target ethnicities (other than AVN/CZVN/CZE) and non-heterosexuals (self-reported) were excluded from the analysis. All participants provided informed consent by clicking on the ‘I agree’ button.

We assessed interrater agreement using intraclass correlation (ICC, 3k; see^132^). Interrater agreement was generally high (ICC for all rater datasets > 0.95; see supplementary Table S1).

### Geometric morphometrics and anthropometric measurements

We analysed facial shape variance using geometric morphometrics. In TpsDig2 software^133^, we manually landmarked each facial image following a predefined^129^ layout of 72 landmarks. Of these, 36 points, true landmarks, denote anatomically and geometrically identical points across faces. Another 36 points, semilandmarks, denote curves between the true landmarks. The semilandmarks are allowed to slide along the denoted curves during shape analysis to minimise the bending energy between corresponding points across faces in a set.

Shape analysis proper was conducted using the Procrustes fit (a generalised Procrustes analysis). The generalised Procrustes analysis was executed using the *gpagen* function of the *geomorph* package^134^. Three variables were computed based on Procrustes residuals: face shape averageness, sexual dimorphism (SShD), and asymmetry. Averageness was computed as the Procrustes distance between the consensus facial configuration and an individual face. It was done separately for male (N = 60) and female (N = 33) facial photographs. To acquire the SShD, we applied the Procrustes fit to pooled male and female shape data (N = 93). Then we projected all faces on an axis connecting male and female averages^108,129,135^. For each face, we extracted a unique score denoting that face’s position along a vector intersecting sex-typical averages. Finally, scores of facial asymmetry were calculated as the sum of squared difference between the original and mirrored (horizontally inverted) version of the same facial configuration^136^.

Higher values of distinctiveness (lower averageness) indicate a greater distance between an individual and the average face. Higher values of asymmetry imply a less symmetrical facial configuration. Higher positive scores of SShD denote more male-like facial shape, while higher negative scores indicate more female-like facial shape.

We also took the body height and weight measurement of each photographed person using calibrated tools. Subsequently, we calculated the body mass index (BMI) as weight (in kilograms) divided by the square of a person’s height (in meters). We recorded the self-reported age of each person.

### Statistical analyses

**Figure 1:**
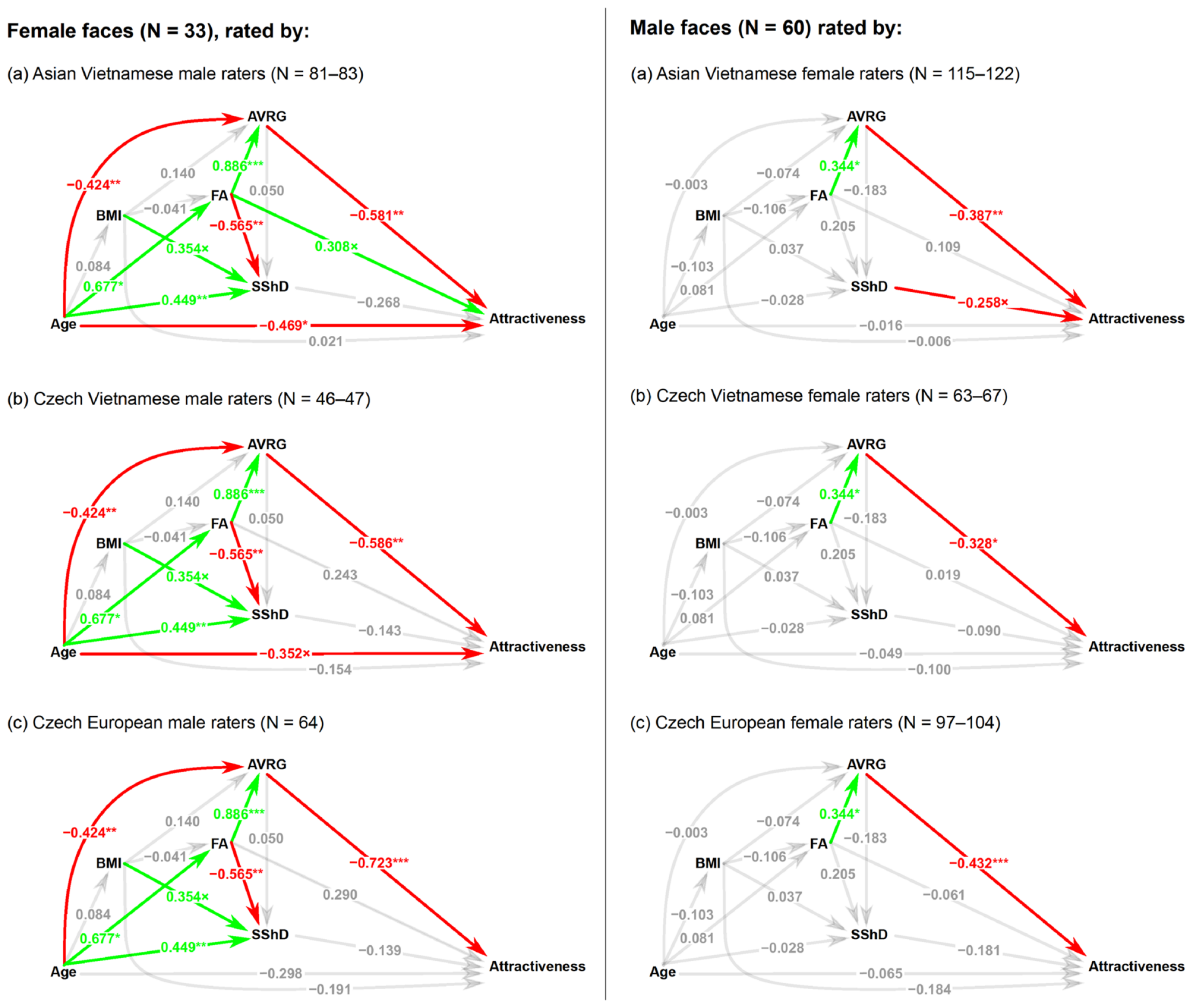
Visualisation of path analyses of correlations between reported age, body mass index (BMI), facial asymmetry (FA), measured averageness (AVRG), sexual shape dimorphism (SShD), and attractiveness. Arrows denote causal directions. Numbers next to paths describe the estimate of regression coefficient of the model with standardised variables. Green arrows denote a positive coefficient, red arrows a negative one. Asterisks represent the level of significance (*p* < 0.05*, *p* < 0.01**, *p* < 0.001***) of partial regression coefficient being non-zero. Gray arrows denote the absence of a significant relationship. Multiplication sign (×) denotes a nonsignificant trend (*p* < 0.1, *p* > 0.05). The higher the SShD value, the more female sex-typical is the facial shape, while lower (negative) values correspond to more masculine facial configurations. The higher the AVRG value, the more distant is the face from the population average (i.e., less average). The higher the FA score, the less symmetrical is the face.

We employed path analyses to trace directed bivariate relationships between attractiveness, measured facial shape (asymmetry, averageness, and SShD), age, and BMI of the photographed stimuli. They were fitted separately for each combination of rater’s origin (European Czech, Czech Vietnamese, Asian Vietnamese) and the sex of the stimulus (see Fig. 1) using the *sem* function from the *lavaan* package for R software^137^.

Following analyses of Pavlovič et al.^128^, we used in our path analyses only directed bivariate associations. Accordingly, attractiveness entered the model as a response variable. It was directly (without mediation by other variables) predicted by age, BMI, averageness, asymmetry, and SShD. Moreover, BMI, facial asymmetry, SShD, and averageness were set as potential mediators of the association between age and attractiveness, because with ageing, the sex-typicality of facial shape might change and facial features could become relatively heavier, less symmetrical, and more distinctive. We also considered the BMI, itself fitted as age-dependent, as a predictor of all other variables. Asymmetry predicted averageness, SShD, and attractiveness. It is likely that faces with more asymmetric configurations would also be less average. Although the effect of asymmetry would probably be mediated by averageness, we also considered a direct path from asymmetry towards perceived attractiveness (see diagram in Fig. 1).

In our previous study^128^, raters from the same three samples (i.e., Asian Vietnamese, Czech Vietnamese, Czech European) rated Czech faces of European origin. In that study, the layout of path analyses was the same as here except that now we added facial asymmetry. Readers may thus be advised to directly compare the results of the two studies to acquire a fuller perspective on Czech–Vietnamese perception of facial attractiveness.

Prior to analyses, all variables were standardised (scaled to zero mean and variance unity). Due to the relatively low number of raters (47–131) and in order to follow the setup of analysis from Pavlovič et al.^128^, we assessed the significance of effects on the standard 5% alpha level based on ‘robust’ *p*-values, meaning *p*-values obtained using the Monte Carlo permutation procedure. We ran the model 10,000-times on randomised data. Then we calculated the distribution of expected regression coefficients for each bivariate association and compared them with coefficients based on the original data. A ‘robust’ *p*-value indicates which portion of distribution density of a given bivariate coefficient is more eccentric than the bivariate coefficient observed in the actual data.

Mixed-effect models were fitted using the lmerTest package for R^138^. We completely specified the varying intercepts and slopes among rater groups, random intercepts for each rater, and random intercepts for each stimulus face. We set the attractiveness ratings as the sole response variable. Age, BMI, averageness, and SShD served as predictors. We included interactions terms between the predictors and rater groups. To do so, we set the varying intercepts and slopes for each of the ‘linear predictor:rater group’ interactions. To compare ratings across the three groups, we used a post hoc test (Tukey HSD). To evaluate contrasts between the groups of raters, we used the *glht* function of the multcomp package for R^139^. We built separate models for male and female stimuli and standardised the predictors prior to analysis. Electronic supplementary material, including code and data, are available online^140^.

### Ethics approval

All sampling and experimental procedures conformed to current institutional, national, and international guidelines as well as the Helsinki Declaration. This study does not include information that could lead to the identification of any particular participant. All procedures were approved by the Institutional Review Board of the Faculty of Science of the Charles University (Protocol Ref. Number 04/2020).

## Results

The results of mixed-effect models are summarised in Tables 1 and 2. Attractiveness ratings of the Asian Vietnamese raters were used as the standard measure of attractiveness for the Vietnamese faces. In the mixed effect models, the predictors of the perceived attractiveness that turned out to be statistically significant followed the statistically significant directed bivariate paths in the path analysis (see the section below). The sole exception was a significant effect of SShD on perceived male attractiveness (β =  − 0.111, SE = 0.054, *p* = 0.041). There was also a significant interaction term between the male part of SShD and a group of raters in the mixed effects model: the effect of SShD was significantly weaker for the Czech Vietnamese (β = 0.072, SE = 0.017, *p* < 0.001) but not for the Czech European female raters (β = 0.018, SE = 0.015, *p* = 0.227; both compared to the Asian Vietnamese as the standard measure). Using post-hoc comparison, we found no similar significant interactions between a rater group and SShD for the female stimuli.

**Table 1:** A summary of results of mixed-effects modelling for men’s faces (female raters).

Random effects								
Groups	Name	Variance	SD	Corr				
ratergroup:rater	(Intercept)	0. 723	0.850					
	Age	0.001	0.026	− 1.00				
	BMI	0.001	0.038	− 0.52	0.52			
	AVRG	0.003	0.053	− 0.66	0.66	0.99		
	SShD	0.001	0.027	− 0.41	− 0.18	− 0.53	0.62	
	FA	0.001	0.015	1.00	− 1.00	− 0.60	− 0.72	− 0.49
Face	(Intercept)	0. 162	0.403					
Residual		0. 684	0.827					

Fixed effects								
	Estimate	SE	t-value	df	*p*-value			
(Intercept)	2.449	0.091	26.924	283.987	0.000	***		
Age	− 0.008	0.053	− 0.132	56.384	0.883			
BMI	− 0.002	0.054	− 0.034	56.646	0.965			
AVRG	− 0.164	0.058	− 2.818	56.944	0.004	**		
SShD	− 0.111	0.054	− 2.034	56.421	0.041	*		
FA	0.042	0.058	0.711	56.254	0.471			
ratergroupCZVN	0.081	0.128	0.633	302.824	0.525			
ratergroupCZE	− 0.106	0.111	− 0.961	306.394	0.340			
age:ratergroupCZVN	− 0.015	0.017	− 0.925	2533.303	0.374			
age:ratergroupCZE	− 0.024	0.015	− 1.690	2677.158	0.102			
bmi:ratergroupCZVN	− 0.043	0.017	− 2.500	546.523	0.010	**		
bmi:ratergroupCZE	− 0.094	0.015	− 6.131	551.490	0.000	***		
avrg:ratergroupCZVN	0.020	0.018	0.923	489.288	0.276			
avrg:ratergroupCZE	− 0.059	0.016	− 3.532	495.932	0.000	***		
sshd:ratergroupCZVN	0.072	0.017	4.127	814.154	0.000	***		
sshd:ratergroupCZE	0.018	0.015	1.150	822.379	0.227			
fa:ratergroupCZVN	− 0.036	0.018	− 1.936	5861.841	0.047	*		
fa:ratergroupCZE	− 0.010	0.016	− 0.547	5940.853	0.546			

Significance levels: ^x^ *p* < .1 *p < .05 **p < .01 ***p < .001.

*CZVN* Czech Vietnamese; *CZ* Czech Europeans; *SShD* face shape sexual dimorphism; *AVRG* distance from the average; *BMI* body mass index; *FA* facial asymmetry.

**Table 2:** A summary of results of mixed-effects modelling for women’s faces (male raters).

Random effects								
Groups	Name	Variance	SD	Corr				
ratergroup:rater	(Intercept)	0. 756	0.869					
	Age	0.012	0.108	0.13				
	BMI	0.022	0.030	− 0.02	0.99			
	AVRG	0.001	0.148	− 0.11	0.97	1.00		
	SShD	0.001	0.037	− 0.85	0.41	0.54	0.61	
	FA	0.012	0.111	− 0.28	− 0.95	− 0.60	− 0.92	− 0.26
Face	(Intercept)	0. 150	0.387					
Residual		0. 667	0.817					

Fixed effects								
	Estimate	SE	t-value	df	*p*-value			
(Intercept)	2.823	0.118	23.948	153.444	0.000	***		
Age	− 0.221	0.110	− 2.014	29.310	0.043	*		
BMI	0.011	0.077	0.143	28.744	0.885			
AVRG	− 0.272	0.097	− 2.815	30.308	0.004	**		
SShD	− 0.128	0.083	− 1.545	28.782	0.123			
FA	0.145	0.135	1.073	29.110	0.282			
ratergroupCZVN	− 0.305	0.161	− 1.898	191.043	0.058	^X^		
ratergroupCZE	− 0.073	0.147	− 0.496	191.042	0.619			
age:ratergroupCZVN	0.039	0.045	0.853	356.860	0.347			
age:ratergroupCZE	0.041	0.041	0.997	356.755	0.275			
bmi:ratergroupCZVN	− 0.091	0.029	− 3.099	1348.108	0.002	**		
bmi:ratergroupCZE	− 0.126	0.027	− 4.717	1348.398	0.000	***		
avrg:ratergroupCZVN	− 0.032	0.045	− 0.711	213.597	0.372			
avrg:ratergroupCZE	− 0.163	0.041	− 3.984	213.573	0.000	***		
sshd:ratergroupCZVN	0.055	0.032	1.719	1466.622	0.084	^X^		
sshd:ratergroupCZE	0.044	0.029	1.534	1466.884	0.125			
fa:ratergroupCZVN	− 0.017	0.054	− 0.337	437.522	0.737			
fa:ratergroupCZE	0.030	0.050	0.592	435.484	0.528			

Significance levels: ^x^ *p* < .1 *p < .05 **p < .01 ***p < .001.

*CZVN* Czech Vietnamese; *CZE* Czech Europeans; *SShD* face shape sexual dimorphism; *AVRG* distance from the average; *BMI* body mass index; *FA* facial asymmetry.

In both male (β =  − 0.164, SE = 0.058, *p* = 0.004) and female faces (β =  − 0.272, SE = 0.097, *p* = 0.004), averageness was a significant predictor of perceived attractiveness. The models also revealed a significant interaction between averageness and groups of raters. In particular, in comparison with Asian Vietnamese standard, the association between averageness and male attractiveness was significantly stronger for Czech European female raters (β =  − 0.059, SE = 0.016, *p* < 0.001). No such difference was seen for Czech Vietnamese female raters (again in comparison to the Asian Vietnamese ratings as the standard measure; β = 0.020, SE = 0.018, *p* = 0.276). For female stimuli, we found a significant interaction between a rater group (Czech Europeans) and averageness (β =  − 0.163, SE = 0.041, *p* < 0.001), suggesting that the attractiveness–averageness association is significantly stronger for Czech European male raters than for the Asian Vietnamese male raters (which formed the standard measure). Once again, this interaction was not significant for Czech Vietnamese male raters.

Post hoc comparisons of selected pairs of rater groups (using the Tukey HSD test) indicated no significant difference between the Asian Vietnamese (AVN), Czech Vietnamese (CZVN), and Czech European (CZE) female raters in their attractiveness assessment of male Asian Vietnamese faces (δ CZVN–AVN = 0.081, SE = 0.128, *p* = 0.800; δ CZE–AVN =  − 0.106, SE = 0.111, *p* = 0.604; δ CZE–CZVN =  − 0.187, SE = 0.132, *p* = 0.333). Similarly, attractiveness ratings of female faces by Asian Vietnamese, Czech Vietnamese, and Czech European male raters did not significantly differ (δ CZVN–AVN =  − 0.305, SE = 0.161, *p* = 0.139; δ CZ–AVN =  − 0.073, SE = 0.147, *p* = 0.873; δ CZ-CZVN = 0.232, SE = 0.169, *p* = 0.354).

The preference for average male faces was significantly different between Czech European and Asian Vietnamese female raters (δ CZVN–AVN =  − 0.059, SE = 0.016, *p* < 0.001) and between Czech European and Czech Vietnamese female raters (δ CZ–CZVN =  − 0.079, SE = 0.019, *p* < 0.001). Czech Vietnamese and Asian Vietnamese preferences for average male faces did not significantly differ (δ CZVN–AVN = 0.020, SE = 0.018, *p* = 0.519). Similar to the case of female faces, preference for faces with a higher averageness was significantly different between Czech European and Asian Vietnamese male raters (δ CZ–AVN =  − 0.163, SE = 0.033, *p* < 0.001) as well as between Czech European and Czech Vietnamese male raters (δ CZ–CZVN =  − 0.131, SE = 0.038, *p* = 0.002). Czech Vietnamese and Asian Vietnamese preferences for average male faces did not significantly differ (δ CZVN–AVN =  − 0.032, SE = 0.036, *p* = 0.644).

Preferences for facial dimorphism in male portraits was statistically different between Czech Vietnamese and Asian Vietnamese female raters (δ CZVN–AVN = 0.072, SE = 0.017, *p* < 0.001) and between Czech European and Czech Vietnamese female raters (δ CZE–CZVN =  − 0.054, SE = 0.018, *p* = 0.005). The difference in preferences for facial dimorphism between Czech European and Asian Vietnamese female raters was not significant (δ CZE–AVN = 0.018, SE = 0.015, *p* = 0.448). Preferences of male raters for facial dimorphism in female faces showed no significant difference between the groups (δ CZVN–AVN = 0.055, SE = 0.032, *p* = 0.194; δ CZE–AVN = 0.044, SE = 0.029, *p* = 0.275; δ CZE-CZVN =  − 0.010, SE = 0.033, *p* = 0.947).

Preferences for facial symmetry did not significantly differ between the compared rater groups neither for male faces (δ CZVN–AVN =  − 0.036, SE = 0.018, *p* = 0.114; δ CZE–AVN =  − 0.010, SE = 0.016, *p* = 0.817; δ CZE-CZVN = 0.026, SE = 0.019, *p* = 0.332) nor for female faces (δ CZVN–AVN =  − 0.017, SE = 0.051, *p* = 0.940; δ CZE–AVN =  − 0.030, SE = 0.047, *p* = 0.802; δ CZE-CZVN = 0.047, SE = 0.054, *p* = 0.661).

### Path analyses

We fitted six path analyses (separately by the sex of the stimuli and the three groups of raters: Asian Vietnamese raters, Czech Vietnamese, and Czech European). The results are summarised in Fig. 1.

In all rater groups, male raters perceived less average female faces as less attractive (β =  − 0.581, *p* = 0.002 for AVN; β =  − 0.586, *p* = 0.001 for CZVN; β =  − 0.723, *p* =  < 0.001 for CZE raters). For Asian Vietnamese male raters and female targets, age was negatively associated with perceived attractiveness (β =  − 0.469, *p* = 0.010). Czech Vietnamese raters tended to perceive younger women as more attractive, too (β =  − 0.352, *p* = 0.062). Asian Vietnamese males tended to perceive higher facial asymmetry as more attractive (β = 0.308, *p* = 0.099). These two moderately strong associations were, however, not significant to the standard significance level (*p* = 0.05). In other words, males from all three ethnical groups preferred more average female faces. Asian and Czech Vietnamese probably used younger age as a cue to attractiveness. Interestingly, Asian Vietnamese raters tended to use facial asymmetry as an attractiveness cue, but in a reverse direction than predicted.

Moreover, in female faces, facial asymmetry was positively associated with the averageness score (β = 0.886, *p* < 0.001) and negatively with the SShD score (β =  − 0.565, *p* = 0.003). Age was positively associated with the SShD score (β = 0.449, *p* = 0.007) and facial asymmetry score (β = 0.677, *p* = 0.018) and negatively associated with the averageness score (β =  − 0.424, *p* = 0.004). It means that younger female faces were more feminine in shape, more symmetrical, and more average. BMI was marginally positively associated with SShD (β = 0.354, *p* = 0.051), meaning that heavier Vietnamese female faces tended to be less feminine in their shape.

Path analyses of male stimuli showed that female raters from all three ethnical groups perceived more average male faces as more attractive (β =  − 0.387, *p* = 0.003 for AVN; β =  − 0.328, *p* = 0.013 for CZVN; β =  − 0.432, *p* < 0.001 for CZE raters). There was a nonsignificant correlation trend between SShD and attractiveness as rated by Asian Vietnamese raters (β =  − 0.258, *p* = 0.050), who perceived more male-like facial shape as slightly less attractive in Vietnamese male facial stimuli. Also in male Vietnamese stimuli, we found a positive association between facial asymmetry and AVRG (β = 0.344, *p* = 0.016).

## Discussion

In our previous study^128^, we showed that Czech raters of both European (CZE) and Vietnamese (CZVN) origin converge on their rating of Czech European faces, while a post hoc comparison (Tukey HSD) showed that Asian Vietnamese raters (AVN) disagree with both the Czech Vietnamese and Czech Europeans. This previous study thus showed that Asian Vietnamese, probably due to the lack of experience with Czech European faces tend to process their facial attractiveness differently. The Czech Vietnamese, on the other hand, converged in their attractiveness assessment with Czech Europeans, which can be interpreted as evidence in favour of the socio-environmental factors hypothesis.

In contrast, the current study, which investigated the perception of Asian Vietnamese faces and used the same three groups of raters (CZE, CZVN, AVN) and the same procedure (Tukey HSD), revealed no significant differences between the rater groups regarding the attractiveness, ascribed to the faces across the three rating groups. While this might be due to insufficient statistical power, the stimuli counts in the two studies were comparable. Moreover, none of the insignificant effects within this study were of a similar size to significant effects in the previous study. We therefore speculate that rather than being the consequence of insufficient statistical power, our results are due to an actual consensus on attractiveness judgements across the three populations. In other words, our findings support the ‘cross-population agreement’ hypothesis and indicate that in the current setup, the environment and ethnicity do not influence facial characteristic attribution across these three unique samples. This is in concordance with findings from other studies, suggesting that various human populations concur on their attractiveness ratings^40,50,140^ Nevertheless, it would also be problematic to generalise and claim that facial attractiveness preferences are universally shared, because several other reports did show disagreement across populations^21,22,39^.

As shown in the previous study^128^, Asian Vietnamese raters showed differences in their attractiveness judgments of Czech European faces, whereas no significant differences in attractiveness judgments among the rater groups were found in the present study. This discrepancy could be attributed to the limited exposure and familiarity of AVN raters with Czech European faces, leading to distinct processing of facial attractiveness compared to Czech Vietnamese and Czech European raters. In the current study with the focus on Asian Vietnamese faces, the familiarity level might have been more balanced across the raters, leading to more consistent results.

All groups of raters based their ratings on similar underlying cues to biological quality. Accurate assessment of these cues requires sufficient experience with Vietnamese faces. While the exposure to members of their families and communities may be enough for Vietnamese raters in both countries, this does not apply for Czech raters of European origin. Their experience must stem from a different source. A credible, albeit speculative, explanation of European Czech’s perceptual expertise regarding Vietnamese faces is that long-term experience with faces of individuals belonging to the Vietnamese minority may have provided them with sufficient knowledge to compensate for the fact that faces of persons of Vietnamese origin are a relatively small part of their ‘visual diet’. Moreover, the coexistence of Czech Europeans and Czech Vietnamese probably led to a substantive weakening of potential causes of cross-populational disagreement on attractiveness rating, which may be due to, e.g., geographic and cultural isolation in conjunction with environmental and socio-economic differences.

It is also conceivable that while the three groups give similar ratings, they base their assessments on slightly different cues^140^. Different characteristics (averageness, sexual dimorphism, youthful appearance) might hypothetically serve as cues to different aspects of biological quality^9,67,83,120^. Still, a person’s overall attractiveness is determined by a wide array of clues^141^. The ‘backup signal’ hypothesis even states that various traits serve as redundant signals to biological quality^142^. To determine whether raters from different groups base their ratings on different facial characteristics which add to the overall attractiveness, we used path analyses, which allowed us to explore potential cross-group differences in a descriptive way, adding up to the results of post-hoc comparison. Moreover, we have also used mixed-effect models with interaction terms between predictor variables and rater groups (entered as fixed effects) to test for possible differences between Asian Vietnamese raters (standard measure) and the other two rater groups.

Across the rater groups, path analyses showed a relatively consistent layout. In general, male raters tended to perceive younger and more average female faces as more attractive. Female raters likewise perceived more average male face as more attractive but did not tend to use age as a cue to male attractiveness.

Shape averageness was thus perceived as an attractive facial characteristic by all three groups of raters with respect to both male and female faces, which is consistent with our previous findings. According to the mixed-effect models, facial averageness was relatively more important for Czech European raters than for Asian Vietnamese raters with respect to both male and female stimuli. No such significant interaction was seen for Czech Vietnamese raters, who preferred facial averageness to a same degree as Asian Vietnamese raters did.

Compared to the two groups of raters of Vietnamese origin, Czech European raters depend more on averageness possibly due to their inability to assess other cues. Facial averageness is a relatively time-stable cue to long-term healthiness^83^, while current health^143^, ageing^144^, and potentially also current fertility^145^ are indicated by different facial traits, which may, however, be hard to assess in unfamiliar faces^140^. A higher level of attention to facial averageness (as a cue to long-term biological quality) in faces belonging to a less familiar ethnicity may thus be a way of partly compensating for limited ability to access other, more current and changeable cues to biological quality. Isolation between groups may even affect the recognition of and preference for average facial traits^73^, but while the Czech and the Vietnamese are culturally distant and visually distinct, they are not mutually isolated (see the Introduction). Alternatively, the preference for facial averageness need not have any adaptive functional explanation and might be just an effect of a link between attraction and statistical typicality^146^.

According to the mixed-effect model, more male-like facial shape (SShD) predicted lower perceived attractiveness of male faces. There was also a significant interaction between the rater’s ethnical group and the effect of SShD on perceived attractiveness, suggesting that the overall negative association between more male-like facial shape and perceived attractiveness is weaker in the Czech Vietnamese than in Czech European or Asian Vietnamese raters. In the path analysis, however, SShD played no significant role in predicting perceived attractiveness for any group of raters except for a marginally significant path for Asian Vietnamese female raters and their assessment of male stimuli. Facial symmetry also played only a limited role: More symmetric female faces tended to be judged by Asian Vietnamese male raters as less attractive, but this effect was not statistically significant in the mixed-effect model and appeared only in the path analysis where various mediation paths were considered. This may potentially point to a limited relevance of measured facial asymmetry as an attractive facial characteristic. While some studies point to the relative importance of facial asymmetry as an attractive trait^87,88^ and a measure of biological quality^77^, other studies reported no association between measured asymmetry and attractiveness or perceived health^89,90^.

Concerning the small effect of the sexually dimorphic facial shape (expressed by SShD) on perceived attractiveness, we can speculate that other sexually dimorphic cues—such as skin coloration or colour contrasts between different facial parts^147^—may play a more important role in attractiveness ratings. In this study, however, they were not considered and no skin colour measurements were taken during the photo sessions.

**Figure 2:**
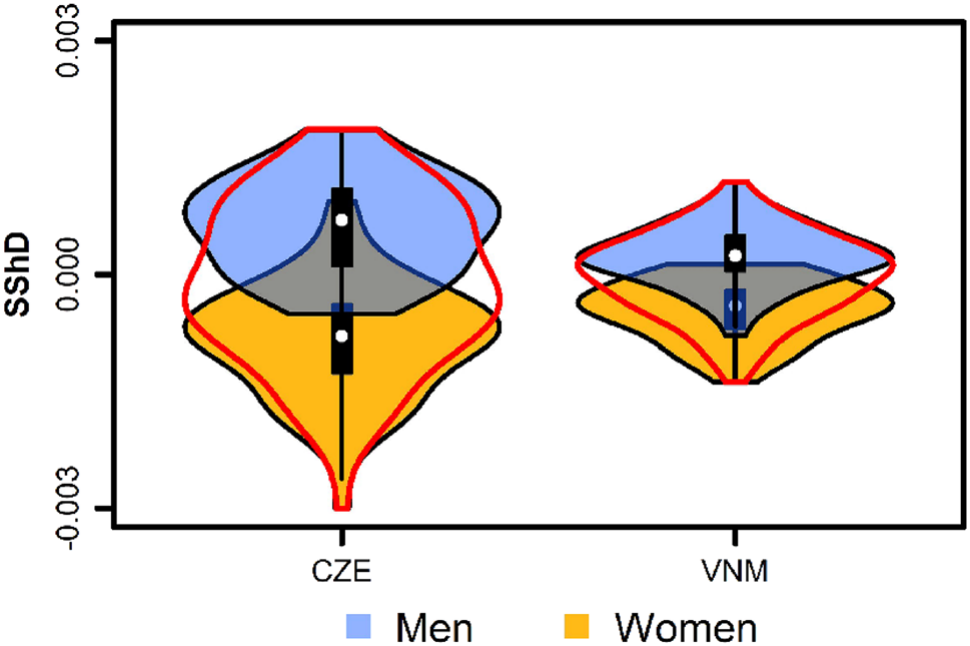
Violin plots comparing the range and variation in sexual shape dimorphism (SShD) between Czech European (CZE) and Asian Vietnamese (VNM) faces. White points indicate medians, black rectangles represent interquartile ranges. The Czech European faces are the same as used in our previous study Pavlovič et al. (2021), Asian Vietnamese faces are identical with those used in the current study.

The magnitude of facial sexual dimorphism varies substantially across populations^129^. Compared to European faces, Asian faces have a lower overall sexual dimorphism^130^ and are generally perceived as more feminine^148^. Cross-group differences in the variance of sexually dimorphic facial shape are shown in Fig. 2, which compares the range and variation of SShD across Vietnamese (current study) and Czech European faces (taken from Pavlovič et al^128^). Sex-typical cues other than SShD might affect the ratings of attractiveness. Moreover, perceivers might use different cues, such as facial averageness, to assess the attractiveness of Vietnamese faces. In fact, unlike the SShD, facial averageness was an important predictor of perceived attractiveness for all groups of raters, which is fairly consistent with the ‘average is attractive’ hypothesis^43,149^ and with our previous study^128^. Some studies state that environmental conditions, such as urbanisation^150^, or society-level measures of economic development and public health^109,151^ also lead to cross-population differences in preferences for sex-typicality. Our data, however, suggest no systematic differences in sex-typicality preference between different groups.

According to the path analyses, age seems to affect the perception of female attractiveness only for Asian Vietnamese and Czech Vietnamese male raters. In similar vein, facial asymmetry and SShD tended to affect the rating only for Asian Vietnamese raters. These trends could imply that age, shape sexual dimorphism, and/or symmetry may be rather ineffective cues to attractiveness rating, especially when it comes to rating the faces of persons from different population. Their effects may raise with increasing experience or long-term exposure to certain distinctive group of faces. Otherwise, perceiver will tend to rely on ‘general’ cues to attractiveness, such as averageness (as discussed in more detail above).

### Limitations

In this study, we used facial photographs, online rating, and frequentist exploratory approach to statistical analysis. While these methods were the most suitable given the ongoing Covid pandemic and relative paucity of prior studies on this or similar subjects (such as preferences regarding own-race faces in a diaspora), it also limits the interpretability of the results. Still, a different approach to such an understudied problem, for instance one based on facial manipulation, could be potentially misleading because any methodological artifacts could easily go unnoticed. Moreover, the methods of geometric morphometrics, which we used to describe facial shape, in conjunction with exploratory multivariate analysis allow for identification of credible associations between facial features and perceived characteristics in highly ecologically valid settings.

The relatively low number of female facial photographs (33 women) lowered the statistical power. On the other hand, the number of observations was sufficient for the use of methods based on linear models (as suggested by^152^) and both the path analyses and mixed-effect models yielded fairly similar results.

The settings of acquisition of facial photographs may also affect study results^131^. We took this into consideration and made sure that all photographs were taken and processed by the same person, with the same camera setting and during a short period of time. Any systematic variation stemming from stimuli collection is thus unlikely to affect the results.

The relatively limited age range of the stimuli group (18–40 years) might reduce the effect of age on the perceived characteristics as compared to general population. But because this age range overlaps with the life stage of choosing a mate and starting a family, it is during this period that attractiveness, a cue to biological quality of a mate, should matter the most.

## Conclusions

The aim of the present study was to investigate several factors known to influence judgements of facial appearance among three groups of raters. All three groups rated the attractiveness ratings of Vietnamese faces similarly, that is, in the current setup the rater’s population and ethnical origin had no major effect on the perceived attractiveness in terms of face shape averageness, asymmetry and sexual dimorphism. We also found that all three groups perceived an Asian Vietnamese face as being significantly more attractive if it had a more average shape. This was as true for men rating female faces as it was for women rating male faces. As a component of facial attractiveness, an average face shape was significantly more important to Czech Europeans than to either group of Vietnamese origin. This highlights the role of averageness as a universally used trait in face perception. Finally, despite some intrapopulational trends in the impact of SShD, facial asymmetry, and age on attractiveness ratings, the three groups did not differ significantly in their overall ratings of facial attractiveness.

These findings suggests that judgements and preferences regarding facial traits are not significantly affected by sociocultural background and geographical context. In other words, our results suggest a universal agreement in ratings across different ethnical groups. However, further work is needed in order to fully explain the influence of environment, visual diet, individual experience, as well as social context on face perception processes, trait attribution, and formation of judgements.

### Supplementary Information

Supplementary Information.

## Data Availability

The dataset and R code is available at https://osf.io/jnpxh/?view_only=821a6a876dbc41ccb4649dad1c0d8e85.
