# Supplementary material for: Congruence in European and Asian perception of Vietnamese facial attractiveness, averageness, symmetry and sexual dimorphism
Source: Sci Rep. 2023 Aug 16;13:13320. doi: 10.1038/s41598-023-40458-1 (PMC10432390; doi:10.1038/s41598-023-40458-1)
Supplement: Supplementary file 1 — Supplementary Information. [file 41598_2023_40458_MOESM1_ESM.pdf]

Congruence in European and Asian perception of Vietnamese facial attractiveness, averageness, symmetry and sexual dimorphism.

Ondřej Pavlovič*, Vojtěch Fiala, Karel Kleisner

*Corresponding author: ondrej.pavlovic@natur.cuni.cz

Supplementary information

Table S1 Inter-rater agreement

	CZ men rating VN women			CZ women rating VN men		
Type	Coeff.	Lower CI	Upper CI	Coeff.	Lower CI	Upper CI
ICC(3,k)*	**0.97**	0.95	0.98	**0.98**	0.97	0.98
	CZVN men rating VN women			CZVN women rating VN men		
Type	Coeff.	Lower CI	Upper CI	Coeff.	Lower CI	Upper CI
ICC(3,k)*	**0.96**	0.93	0.97	**0.95**	0.932	0.97
	VN men rating VN women			VN women rating VN men		
Type	Coeff.	Lower CI	Upper CI	Coeff.	Lower CI	Upper CI
ICC(3,k)*	**0.96**	0.945	0.98	**0.976**	0.967	0.98

*Average, fixed raters ICC

Notes: CZVN – Czech Vietnamese; CZ –Czech Europeans, VN - Vietnamese
